# Dexamethasone regulates gene expression in chondrocytes through MKP-1 and downregulates cholesterol hydroxylases CH25H and CYP7B1

**DOI:** 10.1007/s00011-025-02121-5

**Published:** 2025-11-26

**Authors:** Tiina Lehtola, Antti Pemmari, Elina Nummenmaa, Ida Valjus, Mari Hämäläinen, Teemu Moilanen, Katriina Vuolteenaho, Eeva Moilanen

**Affiliations:** 1 The Immunopharmacology Research Group, Faculty of Medicine and Health Technology, Tampere University and Tampere University Hospital in Wellbeing Services County of Pirkanmaa, 33014 Tampere, Finland; 2 Coxa Hospital for Joint Replacement, Tampere, Finland

**Keywords:** Glucocorticoids, MKP-1, Cholesterol, Osteoarthritis, RNA-seq

## Abstract

**Objective:**

Mitogen-activated protein kinase phosphatase-1 (MKP-1) is an anti-inflammatory enzyme whose expression is increased by glucocorticoids (GCs). MKP-1 dephosphorylates and thereby inactivates mitogen-activated protein kinases (MAP kinases) which are major signaling pathways mediating proinflammatory effects of various extracellular factors to gene expression. In this study, we examined the regulatory effects of the synthetic glucocorticoid dexamethasone on the expression of a panel of genes previously identified as the top 15 critical mediators in the pathogenesis of osteoarthritis (OA). Furthermore, we investigated the hypothesis that MKP-1 is involved in mediating these glucocorticoid-induced effects in chondrocytes.

**Methods:**

The effects of dexamethasone on the interleukin-1β-induced expression of OA target genes were investigated with RNA-seq and quantitative RT-PCR in primary cultured chondrocytes from wild-type and MKP-1 deficient mice, and from OA patients undergoing joint replacement surgery.

**Results:**

Under these conditions, dexamethasone was found to significantly alter the expression of seven out of the 15 OA-related genes including two cholesterol hydroxylases, namely cholesterol 25-hydroxylase (CH25H) and 25-hydroxycholesterol 7-hydroxylase (also known as cytochrome P450 family 7 subfamily B member 1, CYP7B1). Dexamethasone attenuated the interleukin-1β -induced expression of CH25H and CYP7B1 in primary chondrocytes of wild-type mice and in primary human OA chondrocytes, but the dexamethasone effect was absent (CYP7B1) or reduced (CH25H) in chondrocytes from MKP-1 deficient mice. Furthermore, the p38 MAP kinase inhibitor BIRB796 significantly inhibited CH25H expression while the JNK MAP kinase inhibitor SP600125 attenuated CYP7B1 expression in human OA chondrocytes.

**Conclusions:**

In conjunction with previous findings, the current data substantiate the role of MKP-1 as a protective factor in chondrocytes and highlight its potential as a therapeutic target for the treatment of osteoarthritis, because increased levels of cholesterol and its metabolism by CH25H and CYP7B1 are involved in the pathogenesis of OA, particularly in its obesity-associated phenotype.

## Introduction

Osteoarthritis (OA) is the most prevalent degenerative joint disease and one of leading causes of disability worldwide. Its incidence is strongly associated with aging and obesity, both of which are increasing in prevalence globally. OA is a multifactorial disease that affects the entire joint, including the synovium, subchondral bone, and ligaments. However, the most prominent pathological changes are observed in the articular cartilage and its cells, the chondrocytes, which are critically involved in the initiation and progression of the disease [1, 2]. Understanding the molecular mechanisms governing chondrocyte function and cartilage homeostasis is therefore essential for the development of effective disease-modifying therapies [3]. Obesity and metabolic syndrome are strongly associated with OA and for a long time OA was considered to be a result of biomechanical strain. Nowadays it is understood that OA is a (mechano)inflammatory disease, with a complex pathogenesis and various phenotypes [4–6]. Those partially overlapping OA phenotypes include age-related, post-traumatic, obesity-related, metabolic syndrome-associated, erosive, inflammatory and generalized OA [7, 8]. For example, high-fat diet and obesity lead to pro-inflammatory cytokine production and oxidative stress but also altered lipid metabolism in cartilage. Recently, cholesterol metabolism has emerged as an interesting target in OA pathogenesis [2, 4]. The disease progression has been associated with chondrocyte cholesterol uptake and the production of oxysterol metabolites through increased expression of cholesterol hydroxylases, but further work in the area is needed [9].

The management of OA contains physical exercise and weight loss for overweight and obese patients. Commonly used and recommended pharmacological options to treat OA include topical and oral non-steroidal anti-inflammatory drugs and intra-articular glucocorticoid injections which are particularly used in the treatment of OA exacerbations [10–13]. Glucocorticoids have anti-inflammatory and analgesic effects but also carry a risk of various adverse events due to their wide effect on gene expression. Glucocorticoids mediate their effects by binding to the glucocorticoid receptor (GR), which dimerizes and translocates to the nucleus to bind glucocorticoid response elements (GREs), thereby inducing or enhancing the expression of target genes, including those with anti-inflammatory functions [14]. Additionally, glucocorticoids suppress gene expression through various mechanisms, and they inhibit pro-inflammatory transcription factors like Activator Protein 1 (AP-1) and Nuclear Factor kappa B (NF-κB) [15]. The effects of glucocorticoids in regulating cartilage and chondrocyte gene expression have been under investigation.

In OA, IL-1β induces the production of catabolic enzymes and inflammatory factors through mitogen-activated protein kinase (MAP kinase) signaling. Multiple MAP kinases have been characterized, among which p38 and c-Jun N-terminal kinase (JNK) are particularly notable for their roles in mediating immune responses and regulating inflammatory signaling. The MAP kinase activity is tightly controlled through phosphorylation, which serves as key regulatory switch in these signaling cascades. Mitogen-activated protein kinase phosphatase 1 (MKP-1) is a dual-specificity phosphatase that plays a critical role in attenuating MAP kinase signaling by dephosphorylating and thereby inactivating p38 and JNK. Through this mechanism, MKP-1 serves as an important negative regulator of inflammation [16–20]. Interestingly, glucocorticoid responsive element (GRE) has been identified in the promoter region of the MKP-1 gene [20]. Accordingly, we and others have previously shown that glucocorticoids and some other anti-inflammatory drugs enhance MKP-1 expression [16, 18, 21–26]. Moreover, we have shown that the glucocorticoid dexamethasone attenuates the expression of the cartilage degrading enzyme matrix metalloproteinase 13 (MMP-13) in chondrocytes in an MKP-1 dependent manner [18].

In the present study, we aimed to investigate the impact of the glucocorticoid dexamethasone on the expression of genes identified as the top 15 key targets in the OATarget database [27], and to test the hypothesis that these effects are mediated via MKP-1. Gene expression changes were assessed using RNA sequencing and quantitative RT-PCR in chondrocytes derived from wild-type and MKP-1 deficient mice, as well as in cultured human osteoarthritic (OA) chondrocytes. Dexamethasone was found to down-regulate the expression of cholesterol hydroxylases CH25H and CYP7B1 under inflammatory conditions in chondrocytes in an MKP-1 dependent manner. This underlines MKP-1 as a promising treatment target for osteoarthritis.

## Methods

### Animals

MKP-1-deficient (knockout, KO) C57BL/6 mice originally developed in the laboratory of R. Bravo at the Bristol-Myers Squibb Pharmaceutical Research Institute (Princeton, NJ, USA)were utilized in this study [28]. Corresponding wild-type mice were also used. All animal procedures were conducted in compliance with Directive 2010/63/EU on the protection of animals used for scientific purposes and the National Animal Experiment Board approved the study. The 3R principle in the use of experimental animals was followed.

### Mouse chondrocyte isolation and culture

After euthanizing the mice, full-thickness articular cartilage was harvested from their femoral heads, followed by an overnight enzymatic digestion at 37 °C in 5% CO_2_ using Collagenase D (3 mg/mL; Sigma-Aldrich, St. Louis, MO, USA), to isolate chondrocytes, as previously described [29].

The isolated cells (2.0 × 10^5^ cells/mL) were seeded in 24-well plates in Dulbecco’s Modified Eagle’s Medium (DMEM; Sigma-Aldrich) supplemented with 10% fetal bovine serum (Lonza, Verviers, Belgium) and antibiotics penicillin (100 U/mL), streptomycin (100 µg/mL) and amphotericin B (250 ng/mL), all from Gibco/Life Technologies (Carlsbad, CA, USA). Cells were cultured for seven days prior to experimentation. Treatments included interleukin-1β (IL-1β; R&D Systems Europe, Abingdon, UK) with or without dexamethasone (Orion Corporation, Espoo, Finland).

### Human OA chondrocyte isolation and culture

The Ethics Committee of Tampere University Hospital, Finland, approved the study protocol and the study was conducted according to the principles outlined in the Declaration of Helsinki. All participating patients provided written informed consent. Cartilage samples were obtained as surgical waste from osteoarthritis patients undergoing total knee replacement surgery. The cartilage specimens were processed, and chondrocytes isolated as previously described [30]. In brief, articular cartilage was removed aseptically from subchondral bone, cut into pieces, and washed with PBS. The chondrocytes were enzymatically isolated by incubating the cartilage pieces for 16 h at 37 °C in a shaker in the presence of Liberase™ enzyme blend (0.25 mg/mL, Roche, Mannheim, Germany).Chondrocytes (2.0 X 10^5^ cells/mL) were cultured in 24-well plates at 37 °C in 5% CO in DMEM (Sigma-Aldrich) supplemented with 10% fetal bovine serum (Lonza) and antibiotics penicillin (100 U/mL), streptomycin (100 µg/mL) and amphotericin B (250 ng/mL), all from Gibco/Life Technologies, and. In the experiments, the chondrocytes were treated with IL-1β (R&D Systems Europe) alone or together with dexamethasone (Orion Corporation), SP600125 (Sigma-Aldrich) or BIRB796 (Axon MedChem, Groningen, The Netherlands).

### RNA isolation and sample preparation

The culture medium was aspirated at the designated time points, and the chondrocyte total RNA was extracted using the RNeasy Mini Kit (Qiagen, Hilden, Germany), with on-column DNase I treatment to eliminate contamination of genomic DNA. Bioanalyzer system 2100 (Agilent Technologies, Santa Clara, CA, USA) was used to assess RNA concentration and integrity.

### Next-generation sequencing and data analysis

RNA-seq was performed on eight pooled chondrocyte samples from MKP-1-deficient mice and eight pooled samples from corresponding wild-type controls. The llumina NextSeq 500 System was used to sequence the samples with a sequencing depth of 20 M paired-end reads, 75 bp in length at Biomedicum Functional Genomics Unit (University of Helsinki, Finland). FastQC was used to assess read quality [31]. Trimmomatic was used to trim the reads and Spliced Transcripts Alignment to a Reference (STAR) was used to align the reads to reference mouse genome (GRCm38.p6) [32]. Count matrices were prepared with featureCounts [33] program. DESeq2 was used to assess differential gene expression [34].

### Reverse transcription polymerase chain reaction

Total RNA was extracted as described above and reverse transcribed to cDNA using Maxima First Strand cDNA synthesis kit (Thermo Fisher Scientific, Waltham, MA, USA), and PCR was performed using TaqMan Universal PCR Master Mix and the ABI 7500 Real-Time PCR system (Applied Biosystems, Foster City, CA, USA). Mouse CH25H (Mm00515486_s1), mouse CYP7B1 (Mm00484157_m1), human CH25H (Hs02379634_s1) and human CYP7B1 (Hs01046431_m1) were detected using TaqMan® Gene Expression assays obtained from Thermo Fisher Scientific. The in-house primers and probes for human and mouse GAPDH were as previously published [18] and were purchased from Metabion (Martinsried, Germany).

### Statistics

Data was analyzed using GraphPad Prism 10.0.1 (GraphPad Software, Boston, USA) and presented as mean + standard error of the mean (SEM). Statistical significance was calculated using ordinary or repeated measures one-way or two-way ANOVA (analysis of variance), followed by Bonferroni post-test. Differences were considered significant at *p < 0.05, **p < 0.01, ***p < 0.001 and ****p < 0.0001. The fold change comparisons between the genotypes for the RNA-Seq data were derived from log2FC and lfcSE (standard error of log2FC) values obtained from DESeq2. T-test was performed with Bonferroni post-test.

## Results

We utilized the OATargets knowledge base [27] which collects data of potential therapeutic targets to treat OA based on animal models and OA transcriptomics. The effects of dexamethasone on chondrocytes from wild-type and MKP-1 deficient mice were assessed using genome-wide expression analysis (RNA-Seq). The top 15 genes in the OATarget database [27] (Table 1) were explored, and meaningful dexamethasone effect was set at fold change (FC) > |2| and mean expression level in DESeq2-normalized counts more than 20. With these criteria, dexamethasone significantly altered the expression of seven out of the 15 OA-related genes in chondrocytes from wild-type mice, and five of those in cells from MKP-1 deficient mice (Table 2).

**Table 1:** List of all 15 genes analyzed in this study that have been recognized as potential therapeutic targets to treat OA in the OATargets database [23]

Gene	Full name	Function	Role in the pathogenesis of OA
ITGBL1	Integrin subunit beta like 1	Integrin binding	Protective
MT1A	Metallothionein 1A	Metal ion binding	Ambiguous
ADAMTS7	ADAM metallopeptidase with thrombospondin type 1 motif 7	Degradation of extracellular matrix	Detrimental
UCMA	Unique cartilage matrix associated	Bone morphogenic protein binding	Protective
ADAMTS5	ADAM metallopeptidase with thrombospondin type 1 motif 5	Degradation of extracellular matrix	Detrimental
PANX3	Pannexin 3	Regulation of calcium channel activity	Detrimental
NUDT7	Nudix hydrolase 7	Hydrolyzing variety of CoA species	Protective
IL20	Interleukin 20	Inflammation, regulation of hematopoiesis	Detrimental
PHEX	Phosphate regulating endopeptidase X-linked	Regulation of osteogenic cell differentiation	Protective
DMP1	Dentin matrix acidic phosphoprotein 1	Mineralization of bone and dentin	Protective
CH25H	Cholesterol 25-hydroxylase	Cholesterol metabolism	Detrimental
CYP7B1	Cytochrome P450 family 7 subfamily B member 1	Cholesterol metabolism	Detrimental
EPYC	Epiphycan	Regulation of fibrillogenesis	Protective
TNFAIP6	TNF alpha induced protein 6	Extracellular matrix stability and cell migration	Protective
FOXD1	Forkhead box D1	Regulation of transcription factor activity	Protective

The NCBI Gene database (https://www.ncbi.nlm.nih.gov/gene) was used to obtain gene functions and their roles in the pathogenesis of OA are explained as stated by Soul et al [27]

**Table 2:** Dexamethasone regulated the expression on seven out of the top 15 genes in the OATargets database

Gene	WT (IL)	WT (IL+Dexa)	WT FC	WT *p*-value	KO (IL)	KO (IL+Dexa)	KO FC	KO *p*-value	*p*-value for FC comparisons between the genotypes
*Genes downregulated by dexamethasone in wild-type mice chondrocytes*									
CH25H	231	8	− **18.90**	**< 0.0001**	509	98	− **5.13**	**< 0.0001**	**0.001**
TNFAIP6	119	34	− **3.29**	**< 0.0001**	189	43	− **4.23**	**< 0.0001**	0.990
ADAMTS5	5393	1731	− **3.12**	**< 0.0001**	8472	7194	− 1.16	0.0017	**0.001**
CYP7B1	413	197	− **2.07**	**< 0.0001**	412	387	− 1.06	0.5022	**0.001**
*Genes upregulated by dexamethasone in wild-type mice chondrocytes*									
UCMA	1635	15,976	**9.65**	**< 0.0001**	542	5623	**10.41**	**< 0.0001**	0.990
ITGBL1	172	916	**5.21**	**< 0.0001**	150	705	**4.66**	**< 0.0001**	0.990
EPYC	1962	4429	**2.27**	**< 0.0001**	1156	2803	**2.41**	**< 0.0001**	0.990

RNA-Seq analysis was conducted on chondrocytes isolated from wild-type (WT) and MKP-1 deficient (knockout, KO) mice. Cells were treated for 24 h with interleukin-1β (IL-1β, 100 pg/mL), either alone or in combination with dexamethasone (Dexa, 1 μM). From the RNA-Seq dataset, the top 15 genes implicated in OA pathogenesis (as listed in Table 1) were selected for further analysis. Genes were excluded if their expression was not significantly altered by dexamethasone (defined as a fold change [FC] between –2 and 2) or if their mean DESeq2-normalized expression was below 20. P-values for genotype-specific fold changes were adjusted using the false discovery rate (FDR) method. Figures markedly affected (with both FC > 2 in either direction and FDR adj. p < 0.05) are highlighted in bold. Comparative fold changes between genotypes (final column) were derived from DESeq2-generated log2FC and standard error (lfcSE) values. Statistical significance was assessed using a t-test followed by Bonferroni post-test. Statistically significant p-values (p < 0.05) are highlighted in bold

Among the studied OA-related genes, dexamethasone downregulated the expression of cholesterol 25-hydroxylase (CH25H) in both genotypes. In chondrocytes from wild-type mice the fold change was − 18.90, *p* < 0.0001 and in chondrocytes from MKP-1 deficient mice, − 5.13, *p* < 0.0001. The difference between the two genotypes was statistically significant (*p* = 0.001). 25-Hydroxycholesterol 7α-hydroxylase (also known as cytochrome P450 family 7 subfamily B, CYP7B1) expression was attenuated by dexamethasone in chondrocytes from wild-type mice (fold change − 2.07, *p* < 0.0001), but not in chondrocytes from MKP-1 deficient mice (*p* = 0.5022) and the difference between the genotypes was statistically significant (*p* = 0.001). Similarly, the expression of the cartilage-degrading enzyme ADAMTS5 was attenuated by dexamethasone in chondrocytes from wild-type mice (fold change − 3.12, *p* < 0.0001) but the effect was very small in MKP-1 deficient mice chondrocytes (fold change − 1.16, *p* = 0.0017). The difference between the two genotypes was statistically significant (*p* = 0.001), as previously reported [18].

Tumor necrosis factor alpha induced protein 6 (TNFAIP6) was significantly downregulated by dexamethasone in chondrocytes from both wild-type and MKP-1 deficient mice, however there was no significant difference between the genotypes.

In the RNA-Seq analysis, the expression of unique cartilage matrix-associated protein (UCMA), integrin subunit beta like 1 (ITGBL1) and epiphycan (EPYC) in chondrocytes was upregulated by dexamethasone, but there were no significant differences between the genotypes.

**Fig. 1:**
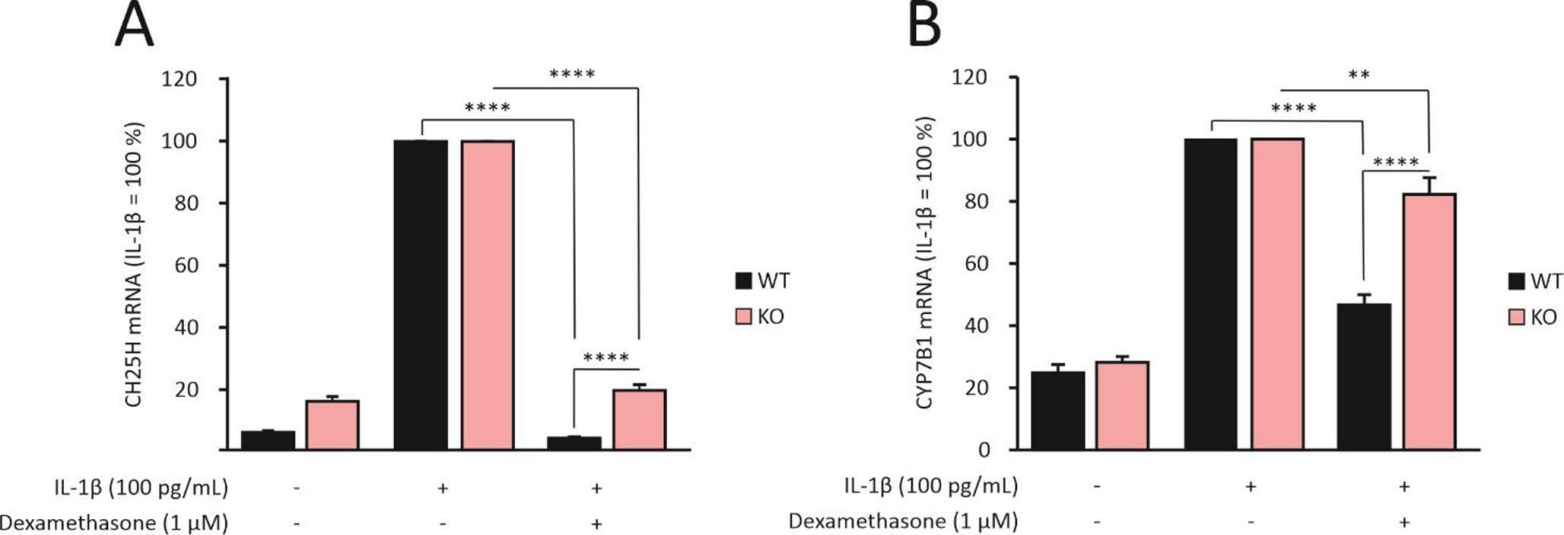
CH25H and CYP7B1 expression was downregulated by dexamethasone in primary chondrocytes from wild-type (WT) mice, and the effect was attenuated in chondrocytes from MKP-1 deficient (knockout, KO) mice. Murine chondrocytes were stimulated with IL-1β (100 pg/mL), either alone or in combination with dexamethasone (1 μM), for 24 h. CH25H (**A**) and CYP7B1 (**B**) mRNA was measured by RT-PCR and normalized to GAPDH. For each genotype, IL-1β-treated samples were set as 100%, and the other values are presented in relation to those values. Data are shown as mean + SEM (n = 8). Statistical analysis was carried out with two-way ANOVA followed by Bonferroni post-test; **p < 0.01, ***p < 0.0001

**Fig. 2:**
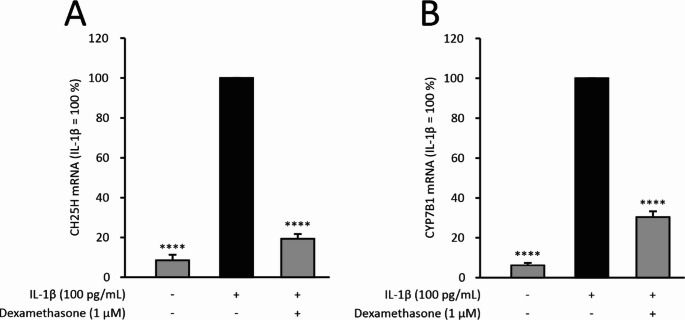
CH25H and CYP7B1 expressions were suppressed by dexamethasone in primary human OA chondrocytes. Chondrocytes were treated with interleukin-1β (IL-1β, 100 pg/mL), either alone or in combination with dexamethasone (1 μM), for 24 h. CH25H (**A**) and CYP7B1 (**B**) mRNA was measured by RT-PCR and normalized to GAPDH. Expression in the IL-1β-treated samples was set as 100%, and the other values are presented in relation to that value. Data are shown as mean + SEM (n = 5). Statistical analysis was carried out with repeated measures ANOVA followed by Bonferroni post-test; ****p < 0.0001

The RNA-Seq results on CH25H and CYP7B1 were confirmed with quantitative RT-PCR (Fig. 1). Consistent with the RNA-Seq results, dexamethasone was found to attenuate IL-1β-stimulated CH25H and CYP7B1 expression in chondrocytes from wild-type mice, but in cells from MKP-1 deficient mice the effect was modestly diminished; in the statistical analysis with two-way ANOVA, the interaction between the genotype and the treatment was statistically significant for both of the cholesterol hydroxylase genes (*p* < 0.0001). This indicates that the dexamethasone effect was reduced in MKP-1 deficient animals and partially dependent on MKP-1 (Fig. 1). In addition, human OA chondrocytes were found to express CH25H and CYP7B1, and their expression was increased by IL-1β (Fig. 2). Like in mouse chondrocytes, dexamethasone significantly inhibited the expression of these two cholesterol hydroxylases in primary human OA chondrocytes when cultured under inflammatory conditions, i.e. in the presence of IL-1β (Fig. 2).

We have previously reported that dexamethasone upregulates MKP-1 expression in both murine and human chondrocytes [18, 35]. Also in the present RNA-Seq analysis, dexamethasone had a minor enhancing effect on MKP-1 expression in IL-1β -treated wild-type mice chondrocytes (FC 1.16, *p* < 0.0001) despite the 24 h time-point, that is after the strongest MKP-1 response (which was one hour in our previous studies) [35].

**Fig. 3:**
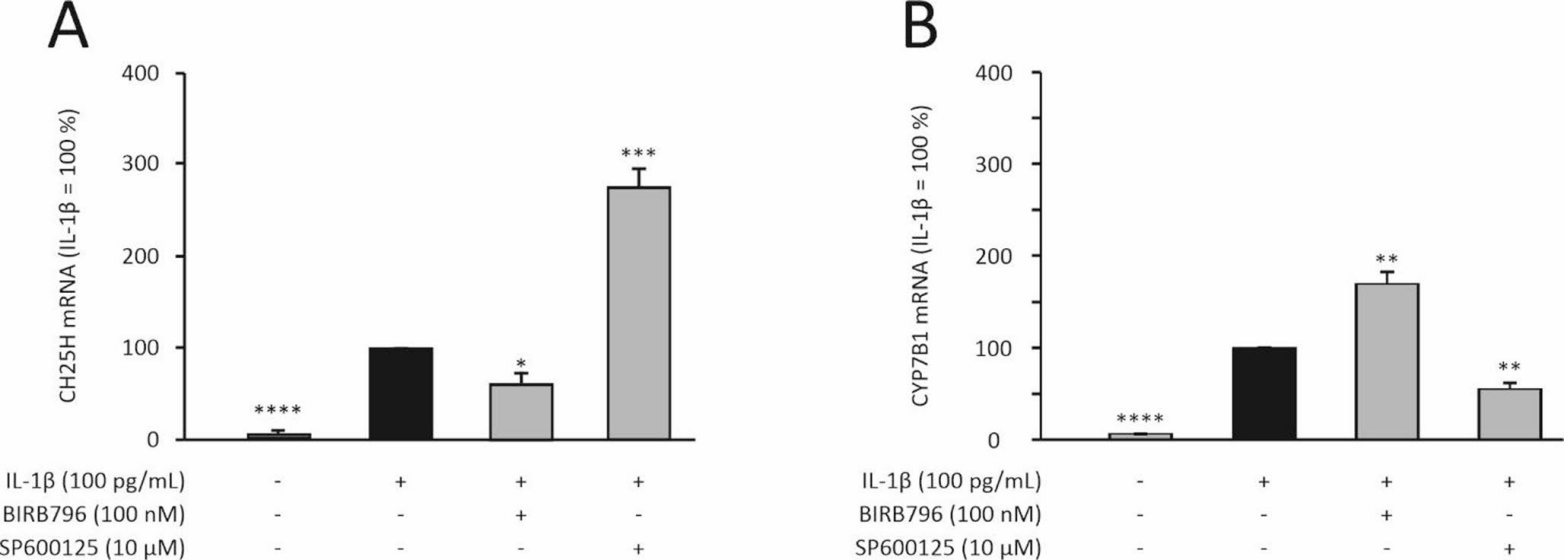
CH25H and CYP7B1 expressions were differently regulated by MAP kinase inhibitors. The primary human OA chondrocytes were treated with IL-1β (100 pg/mL) alone or in combination with the p38 MAP kinase inhibitor BIRB796 (100 nM) or with the JNK MAP kinase inhibitor SP600125 (10 μM) for 24 h. CH25H and CYP7B1 mRNA was measured by RT-PCR and normalized to GAPDH. CH25H (**A**) and CYP7B1 (**B**) expression levels in IL-1β -treated samples were set as 100%, and the other values are given in relation to those values. Data are shown as mean + SEM, n = 6. Statistical analysis was carried out with repeated measures ANOVA followed by Bonferroni post-test; *p < 0.05, **p < 0.01, ***p < 0.001, ****p < 0.0001

MKP-1 is known to inactivate the pro-inflammatory p38 and JNK MAP kinases by dephosphorylation. Since dexamethasone attenuated the expression of CH25H and CYP7B1 through MKP-1, we wanted to further examine if MAP kinase inhibitors have similar effects. Interestingly in human OA chondrocytes, the p38 MAP kinase inhibitor BIRB796 reduced the expression of CH25H, while the JNK MAP kinase inhibitor SP600125 inhibited the expression of CYP7B1 (Fig. 3).

## Discussion

In the present study, we discovered that the interleukin-1β induced expression of the cholesterol hydroxylases CH25H and CYP7B1 in chondrocytes was inhibited by the glucocorticoid dexamethasone, and the effect was mediated by MAP kinase phosphatase 1 (MKP-1). RNA-Seq analysis revealed that dexamethasone significantly downregulated the expression of four genes and upregulated that of three genes out of top 15 genes previously recognized in the OATargets database [27] as potential therapeutic targets for treating OA.

Glucocorticoids are widely utilized anti-inflammatory drugs with broad-spectrum efficacy in the management of various inflammatory disorders [36]. They are commonly administered as intra-articular injections in the treatment of osteoarthritis to alleviate inflammation and pain during exacerbations [10, 11, 13]. The anti-inflammatory actions of glucocorticoids are mediated on one hand through the suppression of proinflammatory genes and, on the other hand through the activation of anti-inflammatory genes, the latter including mitogen-activated protein kinase phosphatase-1 (MKP-1) [36]. Our previous work demonstrated that the glucocorticoid dexamethasone broadly modulates gene expression in osteoarthritic chondrocytes [16], and suppresses MMP-13 expression, an effect partly mediated by the upregulation of MKP-1 [18]. In addition, some anti-inflammatory effects of glucocorticoids in experimental models of inflammatory diseases are mediated through MKP-1 [22–24, 26, 35]. Glucocorticoids have been shown to enhance MKP-1 expression in many cell types including chondrocytes [18, 37], macrophages [38, 39], airway epithelial and smooth muscle cells [40, 41] and endothelial cells [42], and the effect was confirmed also in the present study. The MKP-1 promoter contains a binding site for glucocorticoids (glucocorticoid-responsive element, GRE) as well as for transcription factors such as activator protein 1 (AP-1), nuclear factor κB (NF-κB) and cAMP response element-binding protein (CREB) [20]. Accordingly, aurothiomalate, β2-receptor agonists, and phosphodiesterase 4 (PDE4) inhibitors, and several other anti-inflammatory drugs have also been reported to enhance MKP-1 expression [21, 22]. MKP-1 dephosphorylates and thereby inactivates the pro-inflammatory MAP kinases p38 and JNK [19, 20]. Results presented in this study show that the glucocorticoid dexamethasone alter the expression of seven out of the top 15 ranked genes in the OATargets database [27] in chondrocytes. Moreover, as the RNA-Seq analysis was carried out in both wild-type and MKP-1 deficient mice chondrocytes, we could detect that the glucocorticoid effect on three of these seven genes was mediated through the phosphatase MKP-1.

Epidemiological studies have shown an association between hypercholesterolemia and OA [2, 4, 43]. The original findings by Choi et al. on the role of genes related to cholesterol metabolism—specifically, cholesterol 25-hydroxylase (CH25H) and 25-hydroxycholesterol 7α-hydroxylase (CYP7B1)—in the pathogenesis of OA in experimental models resulted in their inclusion among the top 15 ranked genes in the OATargets database [9, 27]. The CH25H enzyme converts cholesterol to 25-hydroxycholesterol (25-HC) and CYP7B1 metabolizes 25-HC to 7-alpha-25-dihydroxycholesterol (7α,25-DHC) [44]. Retinoic acid-related orphan receptor alpha (RORα) acts as a downstream receptor of these hydroxycholesterols, and it has been found to mediate the OA promoting effects of hydroxylated cholesterols [45]. Osteoarthritic chondrocytes have been shown to contain increased levels of cholesterol and its hydroxylated derivatives because of enhanced cholesterol uptake and CH25H and CYP7B1 expression. Presumably, hydroxylated cholesterol derivatives, in particular, are involved in the pathogenesis of OA, since adenoviral overexpression of CH25H or CYP7B1 in mouse chondrocytes has previously been found to cause experimental osteoarthritis. Moreover, knockdown or knockout of those hydroxylases prevented the development of osteoarthritis [9]. Also, Seo et al. found that increased levels of 25-HC, produced after enhanced CH25H expression, decreased the viability of primary rat chondrocytes [46].

The regulation mechanisms of CH25H and CYP7B1 expression are poorly understood but may offer potential targets for the development of future treatments for OA [9]. We show here that the glucocorticoid dexamethasone attenuates the expressions of CH25H and CYP7B1 in wild-type chondrocytes, but the effect on CH25H was diminished and that on CYP7B1 almost abolished in MKP-1 deficient mice chondrocytes. This downregulating effect of dexamethasone on the CH25H and CYP7B1 expression was also present in human OA chondrocytes. It is of interest that the inhibitory glucocorticoid effect was more intense on CH25H than on CYP7B1 expression in wild-type mice and a similar pattern was observed also in human OA chondrocytes. CH25H and CYP7B1 represent two consecutive enzymatic steps in cholesterol metabolism within chondrocytes, and there may be a functional interplay between them. It is not clear if dexamethasone effects directly on both of the enzymes or if CH25H serves as a primary target, while CYP7B1 could act as a compensatory mechanism. The latter interpretation is supported by the study of Li et al. [45], which showed that miRNA targeting CH25H also led to a subsequent decrease in CYP7B1 expression in chondrocytes. Overall, our results indicate that glucocorticoids have a beneficial and anti-arthritic effect on cholesterol metabolism in chondrocytes.

The regulatory role of MAP kinases on CH25H and CYP7B1 expression remains less studied [47, 48]. Results by Kovács et al. showed, that p38 MAP kinase is involved in mediating the angiotensin II -induced CH25H upregulation in rat vascular smooth muscle cells [49]. The present results in chondrocytes support that finding, since we found that the p38 MAP kinase inhibitor BIRB796 reduced the expression of CH25H. In contrast, the expression of CH25H was increased by the JNK inhibitor SP600125 suggesting that JNK down-regulates CH25H expression in OA chondrocytes under inflammatory conditions. This was somewhat unexpected, and to our knowledge, a novel finding. The mechanism of this effect remains to be investigated but a possible pathway is through transcription factor ATF3: JNK has been reported to promote ATF3 activity, which in turn is known to suppress CH25H expression for instance in macrophages [50]. Unlike the case for CH25H, the JNK kinase inhibitor SP600125 reduced the expression of CYP7B1. This is supported by the previous findings in HEK293 cells where SP600125 suppressed the CYP7B1 promoter activity [51]. While in the same study, the promoter activity was not affected by the p38 inhibitor SB203580 [51]. In the present study, however, p38 inhibition increased CYP7B1 expression in human OA chondrocytes. That may indicate that the effect is conveyed through, for example, RNA stability which is known to be regulated by the p38 MAP kinase [52].

Taken together, the present results show that the inflammatory MAP kinases p38 and JNK regulate CH25H and CYP7B1 expression in chondrocytes in association with OA, and this is a novel finding. MKP-1 is likely to regulate the effect, and we have previously shown that dexamethasone inhibits the phosphorylation of p38 and JNK MAP kinase in an MKP-1 dependent manner [35].

A disintegrin and metalloproteinase with thrombospondin motifs 5 (ADAMTS5) functions as an aggrecanase that cleaves aggrecan, a major proteoglycan in cartilage [53, 54]. ADAMTS7 is also involved in the progression of osteoarthritis since it promotes the breakdown of cartilage oligomeric matrix protein (COMP) [53]. ADAMTS5 seems to be especially important in the pathogenesis of OA, as, for example, its deletion protected joints from cartilage destruction in an OA mouse model [53, 54]. ADAMTS5 and ADAMTS7 are included in the top 15 genes in the OATargets database [27]. In the present study, dexamethasone was shown to attenuate the ADAMTS5 expression in wild-type mice but not in MKP-1 deficient mice chondrocytes, as we have also previously reported [18].

There are some limitations in this study. The results and conclusions presented here are based on mRNA quantification using RNA-Seq and quantitative RT-PCR data obtained from wild-type and MKP-1 deficient mouse chondrocytes and from primary human OA chondrocytes. Further studies are needed to confirm that the detected changes are translated to protein and functional level. The studies with wild-type and MKP-1 deficient mouse chondrocytes strongly suggest that MKP-1 mediates the inhibitory effects of the glucocorticoid dexamethasone on CH25H and CYP7B1 expression. Dexamethasone suppressed the expression of the two cholesterol hydroxylases also in human OA chondrocytes, but direct and selective inhibition of MKP-1 is required to verify its role in mediating these glucocorticoid effects also in human osteoarthritis.

Taken together, the present results show that the expression of several genes implicated in the pathogenesis of OA is significantly regulated by glucocorticoids, and that many of these glucocorticoid effects are mediated through MKP‑1. Among these are recently recognized OA-related cholesterol hydroxylase genes CH25H and CYP7B1, whose expression was downregulated by glucocorticoids in chondrocytes from OA patients and wild-type mice, but the glucocorticoid effect was significantly attenuated in chondrocytes from MKP-1 deficient mice. The role of cholesterol metabolism in the pathogenesis of OA has gained increased research interest and the results presented here link glucocorticoids, MKP-1 and the regulation of cholesterol hydroxylases CH25H and CYP7B1, which is a novel finding. The results reported here, together with earlier studies, support the protective role of MKP-1 in articular chondrocytes under inflammatory conditions and highlight its potential as a therapeutic target for treating osteoarthritis.

## Data Availability

All data supporting the findings and conclusions of the present study are included in the paper.

## Abbreviations

ANOVA: Analysis of variance
AP-1: Activator protein 1
CH25H: Cholesterol 25-hydroxylase
COMP: Cartilage oligomeric matrix protein
CYP7B1: Cytochrome P450 family 7 subfamily B member 1
ELISA: Enzyme-linked immunosorbent assay
FC: Fold change
FDR: False discovery rate
IL: Interleukin
KO: Knockout
MAP kinase: Mitogen-activated protein kinase
MKP-1: Mitogen-activated protein kinase phosphatase-1
MMP: Matrix metalloproteinase
NF-κB: Nuclear factor kappa-light-chain-enhancer of activated B cells
OA: Osteoarthritis
qRT-PCR: Quantitative reverse transcription polymerase chain reaction
RNA-Seq: RNA sequencing
TNF: Tumor necrosis factor
WT: Wild-type
